# ADFSNet: An Adaptive Domain Feature Separation Network for the Classification of Wheat Seed Using Hyperspectral Images

**DOI:** 10.3390/s23198116

**Published:** 2023-09-27

**Authors:** Xin Zhao, Shuo Liu, Haotian Que, Min Huang, Qibing Zhu

**Affiliations:** Key Laboratory of Advanced Process Control for Light Industry (Ministry of Education), School of Internet of Things Engineering, Jiangnan University, Wuxi 214122, China; xinzhao@jiangnan.edu.cn (X.Z.); ls123hi@163.com (S.L.); quehaotian2020@163.com (H.Q.); zhuqib@163.com (Q.Z.)

**Keywords:** domain adaptive, feature separation, wheat seed, hyperspectral images

## Abstract

Wheat seed classification is a critical task for ensuring crop quality and yield. However, the characteristics of wheat seeds can vary due to variations in climate, soil, and other environmental factors across different years. Consequently, the present classification model is no longer adequate for accurately classifying novel samples. To tackle this issue, this paper proposes an adaptive domain feature separation (ADFS) network that utilizes hyperspectral imaging techniques for cross-year classification of wheat seed varieties. The primary objective is to improve the generalization ability of the model at a minimum cost. ADFS leverages deep learning techniques to acquire domain-irrelevant features from hyperspectral data, thus effectively addressing the issue of domain shifts across datasets. The feature spaces are divided into three parts using different modules. One shared module aligns feature distributions between the source and target datasets from different years, thereby enhancing the model’s generalization and robustness. Additionally, two private modules extract class-specific features and domain-specific features. The transfer mechanism does not learn domain-specific features to reduce negative transfer and improve classification accuracy. Extensive experiments conducted on a two-year dataset comprising four wheat seed varieties demonstrate the effectiveness of ADFS in wheat seed classification. Compared with three typical transfer learning networks, ADFS can achieve the best accuracy of wheat seed classification with small batch samples updated, thereby addressing new seasonal variability.

## 1. Introduction

Wheat is a crucial staple crop worldwide, and its quality and yield directly impact global food security. The accurate identification of wheat seed varieties holds immense significance for enhancing the quality of wheat varieties and ensuring the market order [1]. However, accurate identification of wheat seed varieties remains challenging due to factors such as visual similarity between different varieties, lack of distinctive features, and environmental influences on plant growth and development [2,3].

Traditional methods for identifying wheat seed variety rely on time-consuming and subjective visual inspection by experts [4]. The advances in technology such as optical imaging and DNA sequencing are aiding in improving the accuracy of wheat seed variety identification [5]. In recent years, hyperspectral imaging technology has gained significant attention in the field of agriculture for non-destructive and accurate analysis of various crops. However, challenges inherent in hyperspectral data, such as high dimensionality and spectral variability, create significant obstacles for achieving accurate and robust classification results [6,7].

Recently, deep learning combined with hyperspectral images has been widely used in the field of crop seed variety recognition [8,9,10]. Data-driven deep learning models can characterize the relationship between hyperspectral images and crop seed varieties more accurately, but also weaken the generalization ability of the models. The robustness problem of wheat seed variety classification models trained in a single environment is often difficult to solve, and the non-interference from external factors may render the model unsuitable for new environments. In particular, the difference in crop seed varieties originating from different locations, years, and spectral image acquisition processes will greatly impact the accuracy of the model [11,12]. Therefore, finding a strategy that can adapt the model to the new environment with the least cost is the current research focus [13].

Transfer learning is a method for improving the performance of deep learning models on hyperspectral image data by transferring relevant knowledge from a source domain to a target domain, where training and test data exist in different feature spaces [14]. Yang et al. [15] collected hyperspectral images of two different varieties of rice seeds and recorded their germination rate as an index to evaluate seed vitality. First, rice seeds of two different varieties were divided into source domain and target domain according to their varieties. Then, the convolutional neural networks (CNNs) model and residual network (ResNet) model were established on the source domain to predict the vitality index of rice seeds. After fixing the convolution layer parameters of these models, the samples of the target domain were trained to obtain a seed vitality detection model applicable to the target domain. The results showed that introducing a suitable structure with many training samples as feature extractors can increase the generalization ability of hyperspectral image classification models for rice seeds. Wu et al. [16] proposed a method for classifying different crop seeds under limited sample conditions using deep learning model transfer. Inspired by visual geometry group network (VggNet), inception network (InceptionNet), and ResNet structure, three one-dimensional CNNs were developed. The weights of the CNN model based on the source dataset were then transferred to that of the target datasets. Four target datasets (rice, oat, wheat and cotton) were classified via the transfer of these models, achieving accuracies of 95.6%, 99.9%, 80.8% and 83.86%, respectively. At present, research on the transfer learning of deep models in hyperspectral agricultural detection is relatively simple and mostly focuses on fine-tuning parameters [17,18,19]. However, when there is an inconsistency between the source domain and the target domain, the parameters and structure of the pre-trained with the source domain greatly influence the performance of the fine-tuned model.

Generally speaking, the tasks of the source domain and the target domain are the same, and the samples are also similar, so their distribution in the feature space will be relatively close. However, interactions between genotype and environment can lead to changes in wheat seed spectral characteristics, especially due to environmental differences across different years. This interference will cause certain deviations in the distribution of the source wheat seeds and the target wheat seeds in the feature space. These biases are the main reasons that lead to the decline of wheat seed classification performance under different scenarios. Domain adaptation is a research area that focus on transfer learning models in the computer vision field [20]. When the data distribution of the source domain and the target domain is different, but the tasks of both are the same, domain adaptive can be used to apply the model with higher accuracy trained using source domain data to the target domain. It minimizes this deviation by aligning the mapping distribution of the source domain and target domain in feature space, so that the model can obtain a strong generalization ability to cope with new scenarios [21,22].

Most of the domain adaptive transfer learning strategies focus on learning domain feature representation, aiming to learn a feature that is specific to the category but not related to the domain [23,24]. In order to identify transferable components that can be effectively applied from the source domain to improve task performance in the target domain, the adaptive domain feature separation (ADFS) network is proposed in this study. The transfer strategy divides the feature spaces into a shared feature space and two private feature spaces for learning transfer feature representations. The shared features refer to those features that are shared between samples from both domains, and that do not change with variations in varieties and environment. The model uses adversarial training to ensure alignment of the shared feature space. The private features refer to the features that are specific to the source domain and target domain, which are further categorized into class-specific features and domain-specific features by different extractors. The class-specific features are related to the classification task. The domain-specific features only distinguish domains that may not be useful for the target task and could even have a detrimental effect on it. Therefore, these features are excluded from the learning process to prevent negative impacts on the model’s performance in the target domain. In this study, four varieties of wheat seeds from two different years are conducted to verify the validity of the proposed transfer strategy. The performance between the proposed ADFS and three traditional transfer strategies was compared. The primary contributions of this study are (1) proposing a feature separation mechanism to extract domain-irrelevant features; (2) transferring domain-irrelevant features for cross-year wheat seed prediction; and (3) addressing new seasonal variability in wheat seed classification with small batch samples.

The remainder of this paper is organized as follows. Section 2 presents the experimental data, as well as describes the proposed transfer strategy and comparative transfer strategies. Section 3 discusses the results and provides a performance analysis. Finally, Section 4 summarizes the conclusions.

## 2. Materials and Methods

### 2.1. Experimental Samples

In order to investigate the proposed model’s detection ability for wheat seeds from different years, this study collected four different varieties of wheat seeds harvested in 2019 and 2020, namely Bainong4199 (BN4199), Jimai44 (JM44), Weilong169 (WL169) and Zhoumai33 (ZM33). These four types of wheat seed are all semi-winter varieties with strong gluten and high-yield characteristics. All wheat samples were provided by Sinochem Modern Agriculture Co., Ltd. in Beijing, China. For each wheat, 1000 visually undamaged seeds with intact appearance were selected for hyperspectral image sample collection. A total of 8000 hyperspectral images of wheat seeds were collected. The specific information is shown in Table 1. Wheat seeds are produced in Henan and Shandong provinces, and the genetic relationship of part varieties is close.

### 2.2. Hyperspectral Image

**Figure 1:**
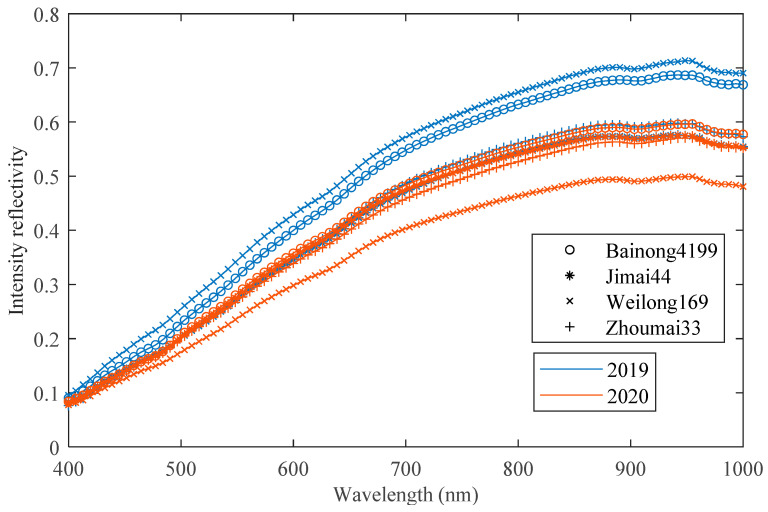
The average spectral curves of four different varieties of wheat seed of 2019 and 2020 year.

This study utilized a line-scan hyperspectral imaging system operating in the visible/NIR range (400–1000 nm) to collect hyperspectral images of wheat seeds. In this study, the spectral resolution of the hyperspectral camera was set to 6.38 nm, the slit range of the spectrometer was 25 μm, the exposure time was set to 250 ms, and the power of the light source was set to 60 w. Each batch of wheat seeds contained 100 samples, and the resulting hyperspectral image data cube had dimensions of 1000 × 1392 × 94, with 94 representing the waveband dimension. After correcting the hyperspectral images, the wheat seed of interest needed to be isolated from the background. For facilitating subsequent modeling, zeros were added around the seed to ensure that each wheat image had a consistent size of 60 × 80 × 94. The details regarding the hyperspectral imaging system and image pre-processing can be found in the paper [25]. Figure 1 displays average spectral curves for four different varieties of wheat seed from 2019 and 2020 year. It is evident that there are significant differences between spectral curves for wheat seeds belonging to same variety but grown in different years.

### 2.3. ADFS Network

Domain adaptive is a novel transfer learning strategy. By mapping inconsistent source domain data and target domain data into a feature space and aligning their distributions in feature space, the knowledge from the source domain can be transferred to the target domain, thereby improving the detection accuracy in the target domain [20].

**Figure 2:**
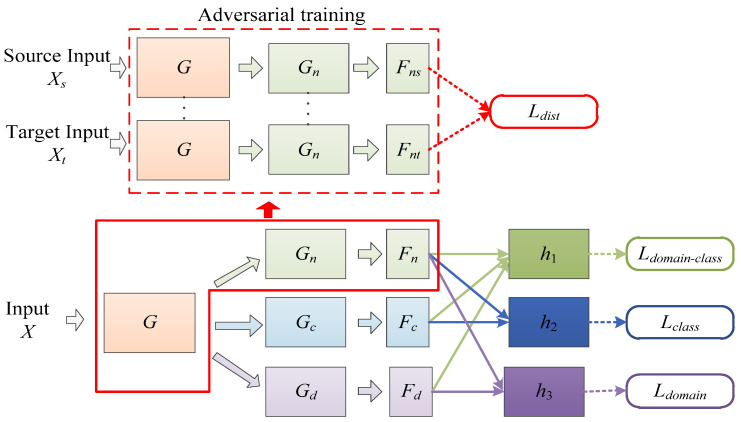
The network structure of ADSF.

**Figure 3:**
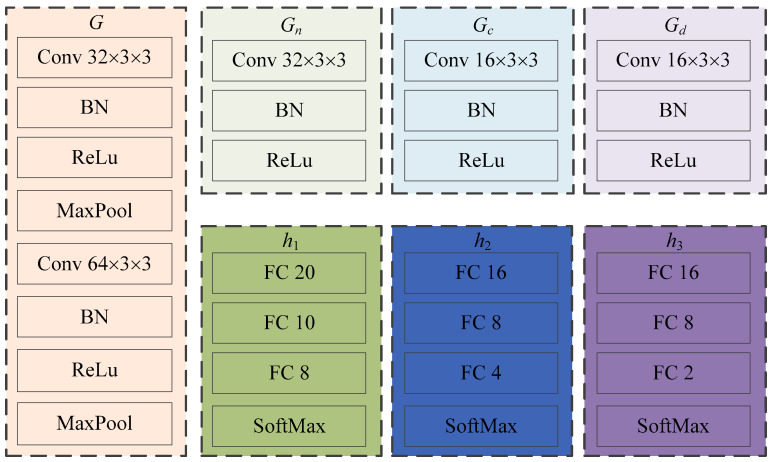
The specific structures of ADFS.

This study introduces the idea of domain feature separation based on domain adaptation to mitigate negative transfer and enhance the model’s detection accuracy. Figure 2 illustrates the network structure of ADFS. In Figure 2, *G*, *G_n_*, *G_c_* and *G_d_* represent feature extraction modules; *F_n_*, *F_c_* and *F_d_* denote the feature map; *h*_1_, *h*_2_ and *h*_3_ are classifier modules. The specific structures of each feature extractor and classifier in the model are shown in Figure 3. Conv 3 × 3 × 3 represents a two-dimensional convolution layer with 32 convolution kernels of size 3 × 3, and other convolution layers follow the same pattern. BN And ReLu indicate batch normalization layer and ReLu activation function layer, respectively. MaxPool symbolizes a maximum pooling layer with a size of 3 × 3 and stride of 2. FC 20 denotes fully connected layer with 20 neurons; remaining fully connected layer is the same. SoftMax represents the SoftMax layer.

In the first stage, the samples are collected for hyperspectral image, image preprocessing, and zero-fill reconstruction to form a dataset consisting of sample data with the same data size. Then, the samples in the dataset are divided into source domain and target domain according to the year, which are input into the feature extractor *G* of the model to obtain mappings of both source and target domain samples in the feature space.

In the second stage, the feature separation module is divided into a shared feature extractor *G_n_* and two private feature extractors *G_c_*, *G_d_*. After abstracting the samples into low-dimensional features, the total features are refined into shared features *F_n_*, class-specific features *F_c_*, and domain-specific features *F_d_*. The shared features *F_n_* refer to the similar features between samples from source domain and target domain. While the class-specific features *F_c_* may shift with domain changes, they contain knowledge helpful for classification tasks within their respective domains. *F_d_* includes information that reflects the difference between the source domain and the target domain. On the other hand, the domain-specific features *F_d_* includes information reflecting differences between the source domain and target domain. They are not helpful to the learning task and may have a negative effect on the target domain task, so the features are not transferred during learning. In the proposed structure, transfer training is performed on *F_n_* and *F_c_* that are domain-irrelevant features.

Since the knowledge represented by the shared features *F_n_* is domain-irrelevant and class-irrelevant, the features from the source domain samples should have a similar distribution to that from the target domain samples. Therefore, cosine distance is used as a loss function to minimize the distribution distance between the shared features from source and the target domains in the feature space. The alignment process of *F_n_* is illustrated in the red box in Figure 2. First, multiple sample pairs are formed by any source domain sample *X_s_* and target domain sample *X_t_*; the sample pair is input into the feature extractor *G* and *G_n_* at the same time. Then, the adversarial training is carried out to calculate the Cosine distances between source sample *X_s_* and target sample *X_t_*, that is the $L_{dist}$ in Figure 2.

(1) $$L_{dist}=1/\frac{G_{n}\left(G\left(X_{s}\right)\right)\cdot G_{n}\left(G\left(X_{t}\right)\right)}{{\left\|G_{n}\left(G\left(X_{s}\right)\right)\right\|}_{2}{\left\|G_{n}\left(G\left(X_{t}\right)\right)\right\|}_{2}}.$$

Finally, the classifiers are constructed by combining the separated features. Since *F_c_* contains features that can distinguish different seed varieties, it is suitable to be used as the input for the class classifier when combined with *F_n_*. Therefore, both are input into the class classifier *h*_2_ in series and trained using the cross-entropy loss function. Similarly, *F_d_* and *F_n_* are connected in series into the domain classifier *h*_3_ and trained using the cross-entropy loss function as the same. In order to further ensure that the refined features have their corresponding feature knowledge, the *F_c_*, *F_d_* and *F_n_* are connected in series and input into domain-class classifier *h*_1_ for domain-class combination prediction and trained as same as *h*_2_ and *h*_3_. The cross-entropy loss function used for $L_{class}$, $L_{domain}$, $L_{domain-class}$ is as follows,

(2) $$\;loss=-\overset{N}{\underset{i=1}{\sum }}y_{i}\log\left(p_{i}\right)$$

where N represents the total number of categories, $y_{i}$ represents the ith bit of the one-hot encoding of the real label of the sample, and $p_{i}$ presents the ith bit of the feature vector output by the feature extractor and Softmax layer of the sample. The total number of digits encoded by sample label one-hot and the total length of the output feature vector of the Softmax layer is equal to the total number of categories of classification tasks. The final loss function is as follows: α, β , γ are weights that control the interaction of the loss terms. The classification loss $L_{class}$ trains the model to predict the output labels we are ultimately interested in. The networks are trained with discovery propagation.

(3) $$\;\;Loss=L_{class}+\alpha \;L_{domain}+\beta \;L_{domain-class}+\gamma \;L{\;}_{dist}$$

### 2.4. Methods for Comparison

There are differences in the feature space of wheat seed classification from different years. The main research directions of feature-based transfer methods include adversarial domain alignment and reconstruction-based domain alignment. Hyperspectral images are 3D images with a spectral dimension. The reconstruction-based domain alignment methods have significant variations and are not applicable to wheat seed classification. Therefore, we chose a representative method of adversarial transfer learning called domain Adversarial Neural Network (DANN) for comparison. Additionally, pre-training and fine-tuning, which are commonly used transfer methods in the spectral domain, were also employed.

**Figure 4:**
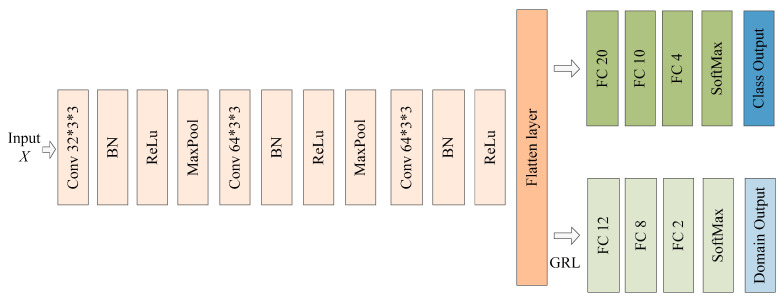
The network structure of DANN.

DANN: DANN was proposed by Yaroslav Ganin et al. [26]. It consists of three parts: a feature extractor, a class classifier, and a domain classifier. The feature extractor and the class classifier can form a complete classification model. The domain classifier used a gradient inversion layer (GRL) to align the features. The model structure is shown in Figure 4.

**Figure 5:**
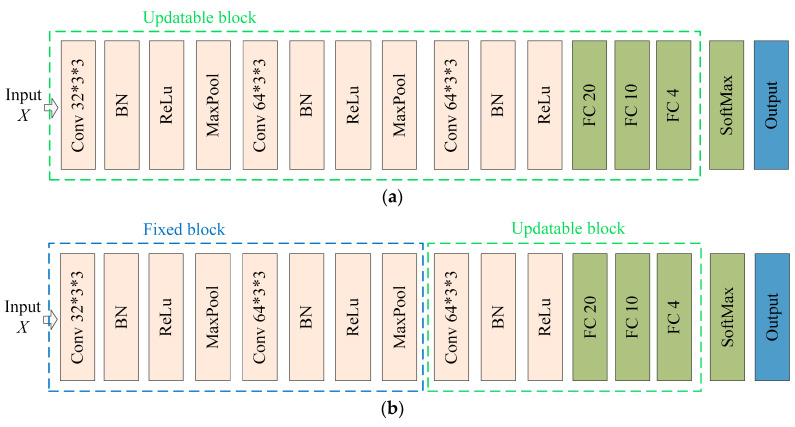
The network structure of (**a**) pre-training and (**b**) fine-tuning.

Pre-training model: The model first used the source domain sample set for pre-training, and then used a small number of samples from the target domain for updating all the parameters so as to transfer the knowledge from source domain to target domain. The model structure is shown in Figure 5a, and the updatable block are framed in green.

Fine-tuning model: The method was similar to the pre-training model, as it also utilized the source domain samples for training. In contrast, the parameters of the shallow feature extraction layer of the convolutional neural network are frozen. Then, a small number of samples from the target domain are used for updating to achieve the purpose of transfer learning. The model structure is shown in Figure 5b. In addition to the updatable block in green box, there are also fixed block in blue box.

The pre-training model and fine-tuning model are based on the same initial CNN that was trained using the source domain samples. The updatable block represents the modules initialized with parameters from the initial model and then updated using target domain samples. The fixed block represents the modules with fixed parameters loaded from the initial model.

## 3. Results and Discussion

In order to enhance the robustness of the detection model in the open environment, this study proposed a novel transfer strategy called ADFS. The wheat seeds harvested in two different years were used as experimental samples. The proposed ADFS was compared with three common transfer methods, and experiments were carried out for transferring from 2019 to 2020 as well as from 2020 to 2019.

### 3.1. Performance Analysis of CNN Model

The structure of initial CNN model is shown in Figure 5. The model is trained using adaptive moment estimation (Adam) with a learning rate lr of 1.5 × 10^−3^, a gradient decay rate $\beta_{1}$ of 0.88 and a gradient squared decay rate $\beta_{2}$ of 0.95. The batch size is 400 and the epoch is 200. The 2019 and 2020 wheat seed samples were randomly divided into training sets and test sets according to 4:1, 1:1 and 1:4, respectively. At the same time, wheat seeds from different years were used as training and test sets, respectively.

The comparison of classification results is summarized in Table 2. Regardless of the data from 2019 or 2020, the best classification results were obtained when 80% of samples were used for training, with accuracies exceeding 94% in both cases. As the training sample size decreases, the classification accuracy also decreases. When only 20% of samples were used for training, the classification accuracies remained above 84%. It indicates that the data distribution was consistent in the same year. The classification accuracy of WL169 is consistently higher than that of the other three wheat seeds in the same scenario prediction. A probable explanation is that WL169 is a wheat variety bred in Shaanxi province, which has strong drought resistance ability. It has slightly different quality characteristics from the other three wheat seed varieties.

Interestingly, when wheat seeds from different years were used for cross-year prediction, the classification accuracies were less than 50%. The poor results were due to inconsistent data distribution in different years. It is difficult to meet the detection requirements by relying solely on the generalization performance of the CNN model itself. When using samples from the source domain, the model cannot learn the feature information of the target domain. It is apparent that WL169 has the worst classification accuracy in cross-year prediction. As can be seen from Figure 1, the average spectral curve of WL169 was significantly different between 2019 and 2020. The difference between cross-year samples leads to a decrease in prediction accuracy. The greater the difference, the worse the prediction accuracy.

### 3.2. Generalization Performance Analysis of the Transfer Learning Models

There were two years of wheat seed samples, and cross-year predictions for different years were compared. In the cross-year predictions, the source target samples were randomly divided into training sets and test sets according to 4:1. The 20% of the target samples with labels were used to update the model for pre-training and fine-tuning models. For DANN and ADFS, 10% of the target domain samples with labels and 10% of the target domain samples without labels were used to optimize the model parameters; the remaining 80% of the target domain samples were tested. The parameter λ of GRL in DANN was set to 1. The parameters α, β , γ of ADFS were set to 1. The other settings are consistent with the CNN model in Section 3.1.

The comparison of cross-year predictions output by different transfer learning models is shown in Table 3. After updating with small batch samples, the pre-training model and fine-tuning model can improve the classification accuracy of wheat seed varieties. However, the overall performance is worse than that of two transfer learning models with domain adaptation. Comparatively speaking, the fine-tuning model is poor. The only difference between the pre-training model and the fine-tuning model lies in whether the parameters of the shallow feature extraction layer are frozen during training. The fine-tuning model with the parameters of the shallow feature extraction layer frozen slightly underperforms compared to the pre-training model, indicating that the original shallow feature extractor is not suitable for the new scenario, thus reducing the overall classification ability of the model.

By introducing domain adaptive, the DANN and ADFS can achieve more than 90% classification accuracy after semi-supervised learning. At the same time, target domain labels are used less than the pre-training and fine-tuning model. The core of the adversarial domain adaptive method adopted by DANN and ADFS is to learn a good enough representation to reduce the difference of different data distributions at the representation level. The proposed ADFS model combines the idea of domain self-adaptation, uses different modules to extract class-specific features, and refines the distribution difference between the source domain and target domain. Adversarial training is used to ensure that the shared parameter spaces are aligned. After the feature is refined, some features that will produce negative transfer and affect the classification effect of the model in the target domain are discarded. Overall, ADFS outperforms DANN in terms of accuracy for wheat seed classification. The transfer performance of ADFS with 10% labeled samples and 10% unlabeled samples is better than 50% of the target sample training model.

**Figure 6:**
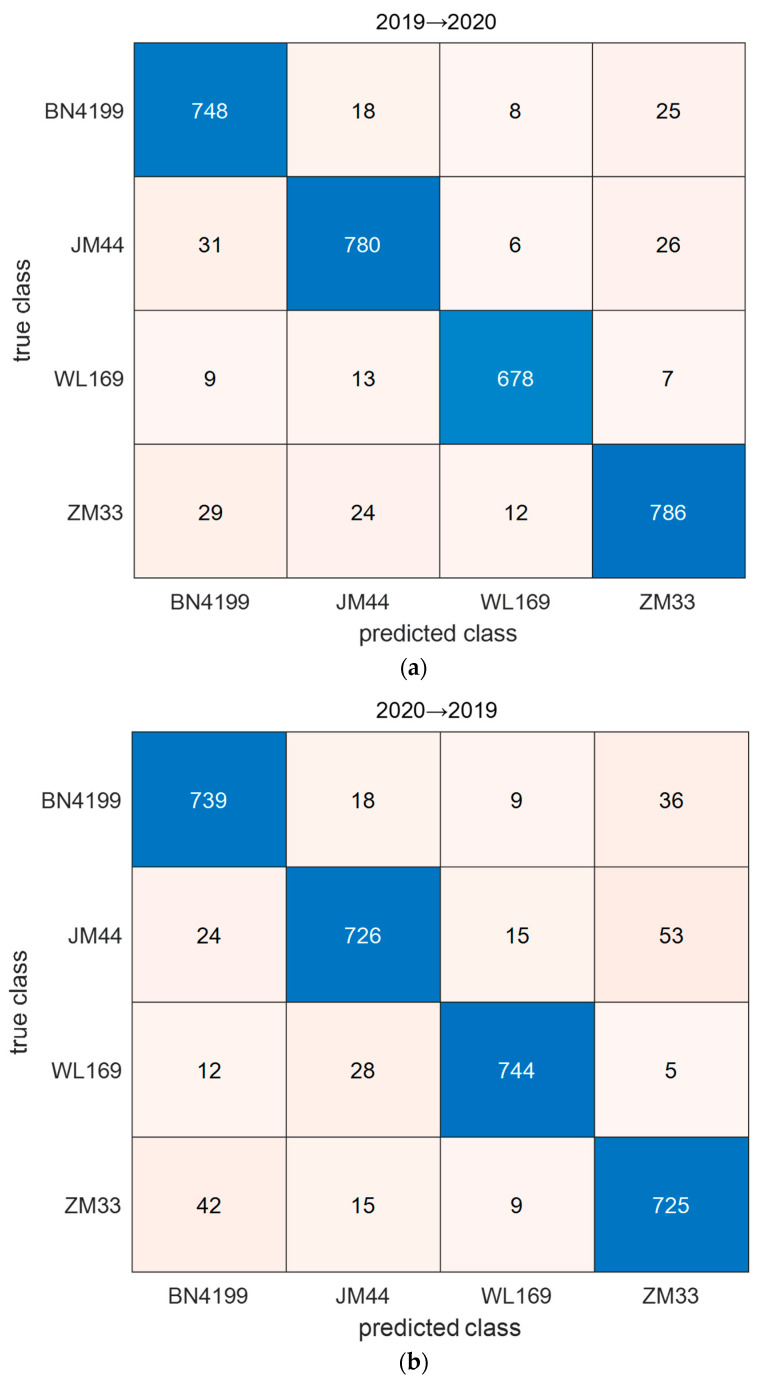
The confusion matrix of the classification results for the target test set by ADFS: (**a**) transfer from 2019 to 2020; (**b**) transfer from 2020 to 2019.

The confusion matrix of the classification results for the target test set by ADFS is shown in Figure 6. As can be seen from the figure, there are more samples misclassified by BN4199 and ZM33 with each other. This result may be related to genetic relationship because BainongAK58 was used to prepare crosses for BN4199 and ZM33. The recall and precision of WL169 are the best among all wheat seed varieties. According to Figure 1, what stands out is the difference between the spectral curve of WL169 and that of the other varieties. This difference may have a positive influence on the transfer results.

**Figure 7:**
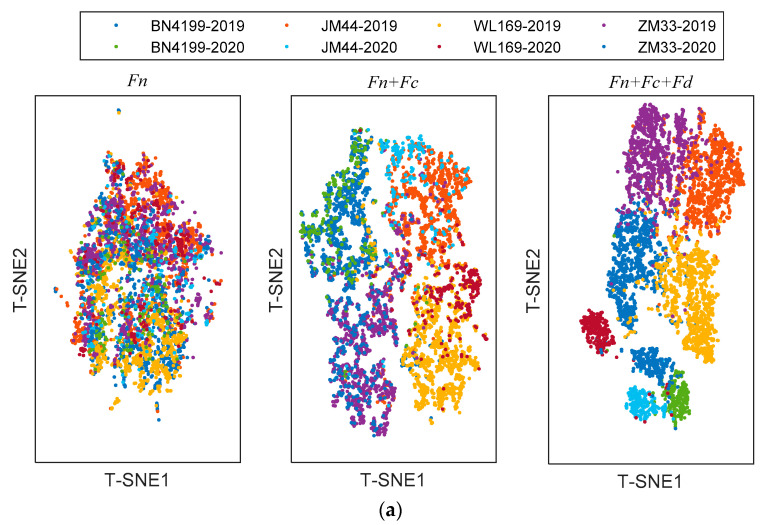

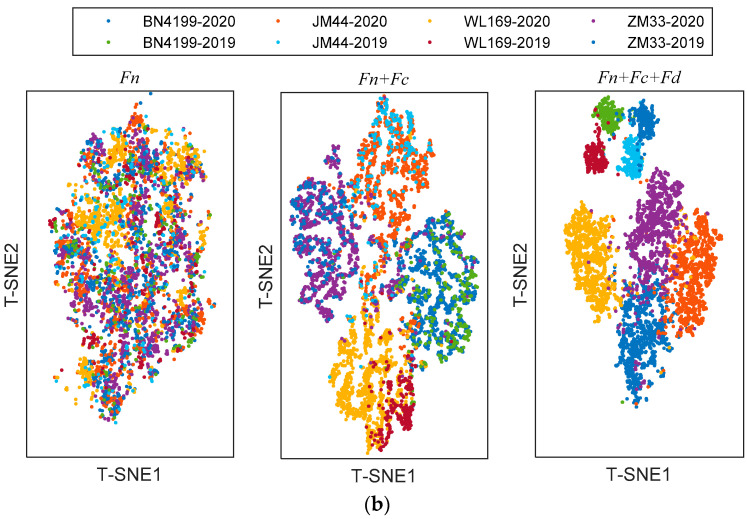
The visualization of the refined features in ADFS via T-SNE: (**a**) transfer from 2019 to 2020; (**b**) transfer from 2020 to 2019.

The shared module captures some shared features across scenarios, the private module captures class-specific features. In order to provide richer judgment for the network, T-Distribution Stochastic Neighbour Embedding (T-SNE) [27] was used to visualize features extracted with different modules. T-SNE can capture complex manifold structures of high-dimensional data and intelligently map multidimensional data to two-dimensional data. The visualization results with different refined features of testing samples are shown in Figure 7. Since this is a classification task, the features are distributed in clusters. When clusters do not overlap and cluster spacing is larger, the extracted features are more effective. The left subgraph is a visualization of the shared features *F_n_*, which are alignment features across scenarios. It is apparent from this subgraph that the shared features are jumbled together and do not shift in distribution with the change in the scenario. The middle subgraph is a visualization of the combined shared features *F_n_* and class-specific features *F_c_*; these features are input into the class classifier. These features are domain-irrelevant, so the source domain and target domain cannot be distinguished. However, there is a difference between different categories. After adding domain-specific features *F_d_*, the visualization of the features is shown in the right subgraph. Samples of different domains and different categories can be well clustered together. However, the cluster is relatively scattered and some cluster boundaries overlap.

### 3.3. Stability of the Transfer Learning Models

**Figure 8:**
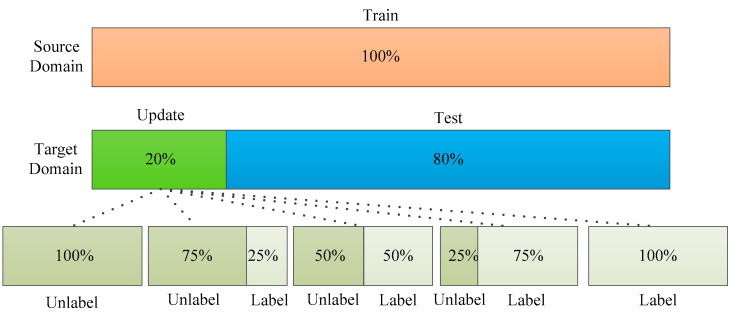
The samples division with different labeled sample proportions.

In order to further study the influence of labeled sample proportion on the stability of the ADFS, experiments of ADFS and DANN with different proportion samples were conducted in this section. The sample division is shown in Figure 8. The proportion of samples for updating is 20% of the total number of target samples; the remaining 80% of the target domain are the test samples. The updated samples are further divided into labeled and unlabeled samples. The sample proportion interval of labeled target samples is 25% of the updated samples, which is also 5% of the target domain samples.

**Figure 9:**
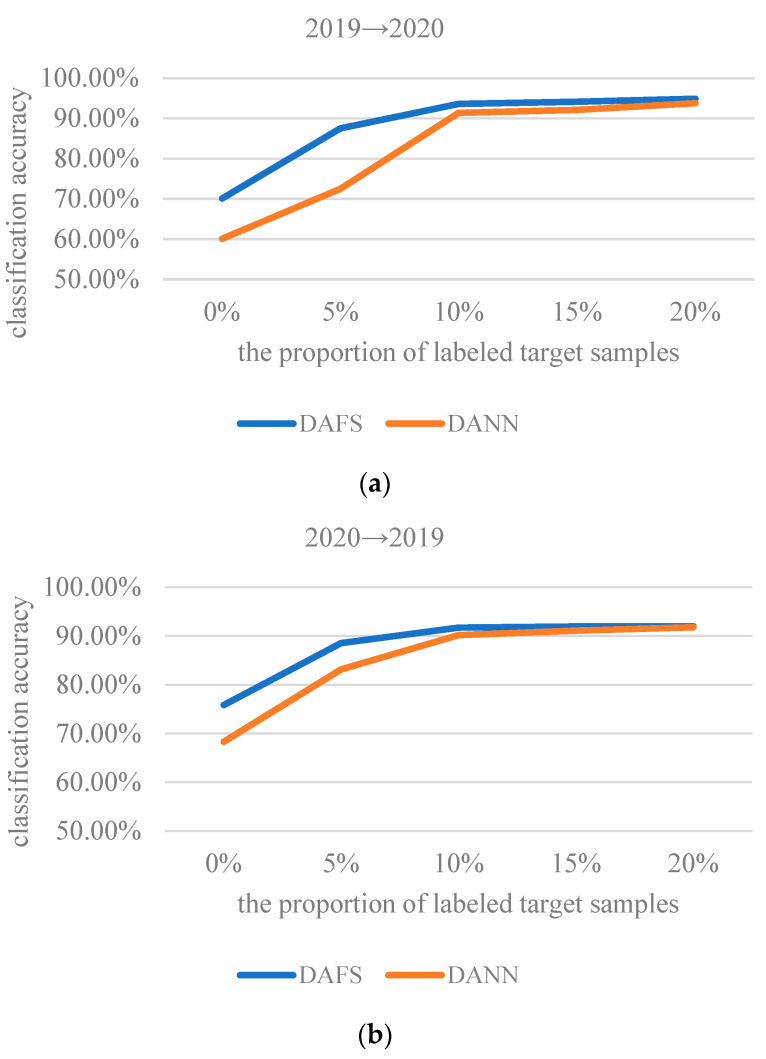
The classification results of wheat seed varieties after adding different proportions of labeled target samples: (**a**) transfer from 2019 to 2020; (**b**) transfer from 2020 to 2019.

The classification results of wheat seed varieties after adding different proportions of labeled target samples are shown in Figure 9. The use of 0% samples means that the model was trained using source domain samples and unlabeled target domain samples. When the added target domain samples are unlabeled, the loss $L_{domain-class}$ and $L_{class}$ is applied only to the source domain.

From Figure 9, we can see that ADSF outperforms DANN in terms of accuracy for wheat seed classification. When fewer labeled target samples were added, the dominance of ADSF was more obvious. What stands out in the figure is that the classification accuracy of unsupervised ADSF is much higher than that of the not-transfer model across scenarios. As the number of labeled samples increases, the classification accuracy increases, and then the later changes are slow. The classification accuracy with 10% labeled samples and 10% unlabeled samples is close to that with 20% labeled samples. It shows that the features conducive to wheat seed classification can be well acquired through the proposed feature separation mechanism, and the features can also be well aligned under the action of a small number of labels. It further demonstrates that the ADSF has good generalization and classification ability for the wheat seed varieties.

### 3.4. Ablation Experiment

**Figure 10:**
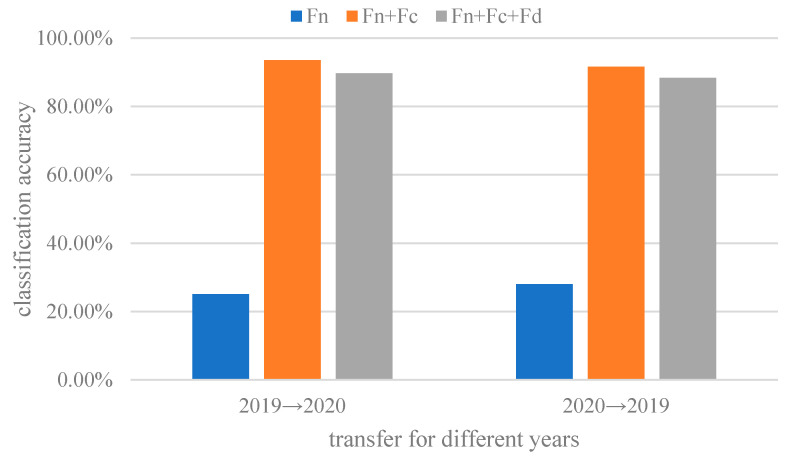
The classification results of wheat seed varieties for different feature sets.

The feature extraction of the ADFS network is carried out through three different modules, which respectively extract shared features class-specific features and domain-specific features. In order to further verify the effectiveness of feature separation and extraction, ablation experiments were performed. The ablation experiment compares the classification results of different separated feature sets, using only the aligned shared feature *F_n_* and all feature set *F_n_* + *F_c_* + *F_d_*. The classification accuracies are shown in Figure 10.

The shared feature *F_n_* constructed in the ASDF network does not include features of classes and domains. Therefore, the classification accuracy using *F_n_* is the worst. As shown in Figure 10, combining shared feature *F_n_* and class-specific *F_c_* can obtain the best classification accuracy. Further domain-specific feture *F_d_* is added and the results become worse. It is precisely due to that *F_d_* is useless for classification, thus causing negative transfer to reduce the classification accuracy.

## 4. Conclusions

In order to address the issue of hyperspectral data transfer for wheat seeds from different years, an ADFS network based on shared and private features is proposed. The network differentiates the feature space into a shared feature space that captures features shared across domains and two private feature spaces that capture features unique to each domain or each variety. After differentiation, the effect of reducing negative transfer is achieved by removing the domain-specific features. Wheat varieties from two different years are used for experimental verification. The results demonstrate that ADFS achieves a classification accuracy of 93.51% and 91.69% when a small number of target domain samples is added. Compared to fine-tuning, pre-training, and DANN approaches with the same target domain sample size, ADSF performs the best in terms of cross-year classification accuracy. The overall results showed that the ADSF network was feasible and could provide accurate cross-year wheat seed variety identification, which would help to develop a system for quality detection of wheat seed under different scenarios.

This study focused on developing a transfer learning network to identify wheat seeds to address data drift across different years. However, the ADSF network still requires a small number of labeled samples for cross-year transfer. In order to better adapt to practical cross-year prediction, future research will concentrate on the unsupervised transfer network with high accuracy.

## Figures and Tables

**Table 1:** Wheat seed details.

Wheat Variety	Producing Area	Genetic Relationship
Bainong4199 (BN4199)	Henan	BainongGaoguang3709F2/BainongAK58
Jimai44 (JM44)	Shandong	Ji954072/Jinan17
Weilong169 (WL169)	Henan	Shanmai94/Xinong822
Zhoumai33 (ZM33)	Henan	Zhengmai366/BainongAK58

**Table 2:** Comparison of classification results of wheat seed varieties under different sample divisions.

Train Set	Test Set	Classification Recall (%)				Average Accuracy
		BN4199	JM44	BN4199	JM44	
80% of 2019	20% of 2019	95.92	94.47	99.05	92.78	95.63
50% of 2019	50% of 2019	91.93	86.90	95.56	85.45	89.95
20% of 2019	80% of 2019	84.87	78.71	90.91	78.88	83.30
100% 0f 2019	100% of 2020	54.20	53.10	15.00	51.50	43.45
80% of 2020	20% of 2020	94.00	91.96	98.98	93.66	94.63
50% of 2020	50% of 2020	87.40	88.93	96.79	92.95	91.55
20% of 2020	80% of 2020	84.22	76.51	95.09	84.84	85.13
100% 0f 2020	100% of 2019	62.90	38.10	19.30	64.20	46.13

**Table 3:** Comparison of transfer learning model performance on different years of wheat seed varieties classification.

Transfer Scenario	Transfer Learning Model	Classification Recall (%)				Average Accuracy
		BN4199	JM44	WL169	ZM33	
2019→2020	No transfer	54.20	53.10	15.00	51.50	43.45
	ADFS	93.62	92.53	95.90	92.36	93.50
	DANN	93.40	87.48	94.14	90.50	91.38
	Pre-training	87.09	88.38	94.97	85.70	89.03
	Fine-tuning	88.39	87.99	89.34	88.15	88.91
2020→2019	No transfer	62.90	38.10	19.30	64.20	46.13
	ADFS	92.14	88.75	94.30	91.66	91.69
	DANN	90.85	88.86	91.69	89.22	90.16
	Pre-training	85.35	90.89	91.12	82.49	87.38
	Fine-tuning	86.88	84.59	86.99	85.00	85.88

## Data Availability

No new data were created or analyzed in this study. Data sharing is not applicable to this article.
